# Disparity-driven vs blur-driven models of accommodation and convergence in binocular vision and intermittent strabismus

**DOI:** 10.1016/j.jaapos.2014.08.009

**Published:** 2014-12

**Authors:** Anna M. Horwood, Patricia M. Riddell

**Affiliations:** a Infant Vision Laboratory, School of Psychology & Clinical Language Sciences, University of Reading, United Kingdom; b Orthoptic Department, Royal Berkshire Hospital, Reading, United Kingdom

## Abstract

**Purpose:**

To propose an alternative and practical model to conceptualize clinical patterns of concomitant intermittent strabismus, heterophoria, and convergence and accommodation anomalies.

**Methods:**

Despite identical ratios, there can be a disparity- or blur-biased “style” in three hypothetical scenarios: normal; high ratio of accommodative convergence to accommodation (AC/A) and low ratio of convergence accommodation to convergence (CA/C); low AC/A and high CA/C. We calculated disparity bias indices (DBI) to reflect these biases and provide early objective data from small illustrative clinical groups that fit these styles.

**Results:**

Normal adults (n = 56) and children (n = 24) showed disparity bias (adult DBI 0.43 [95% CI, 0.50-0.36], child DBI 0.20 [95% CI, 0.31-0.07]; *P* = 0.001). Accommodative esotropia (n = 3) showed less disparity-bias (DBI 0.03). In the high AC/A–low CA/C scenario, early presbyopia (n = 22) showed mean DBI of 0.17 (95% CI, 0.28-0.06), compared to DBI of −0.31 in convergence excess esotropia (n=8). In the low AC/A–high CA/C scenario near exotropia (n = 17) showed mean DBI of 0.27. DBI ranged between 1.25 and −1.67.

**Conclusions:**

Establishing disparity or blur bias adds to AC/A and CA/C ratios to explain clinical patterns. Excessive bias or inflexibility in near-cue use increases risk of clinical problems.

Asymptomatic binocular vision requires integration of angles of deviation, refractive error, and accommodation and convergence to images moving in depth. Atypical accommodative convergence to accommodation (AC/A) ratios characterize a few but not all diagnoses. Complex theoretical models have been developed to explain binocular control, involving feedback loops from accommodation and vergence in combination with tonic and phasic inputs^1^, ^2^, ^3^, ^4^ but do not easily relate to clinical characteristics.

Our research has focused on naturalistic vergence and accommodation to the main cues available in any stimulus moving in depth; blur disparity and proximity (including looming and monocular cues such as size, motion, and awareness of nearness); and the relative weighting between cues.^5^ Two main observations have emerged from these studies supported by earlier work of others.^6^, ^7^, ^8^, ^9^ First, although blur, disparity, and proximity can all drive responses, convergence and accommodation are better when the subject is viewing binocularly, that is, disparity is available ; blur and, particularly, proximity cues drive weak near responses when disparity is absent. Second, variability is normal; some individuals respond to all cues, whereas others strongly favor one.^10^ The purpose of this study was to present evidence in support of a conceptual model that suggests that individual biases in near-cue use predict clinical characteristics. Our model encompasses most clinical diagnoses where bifoveal binocular vision is preserved (intermittent strabismus, heterophoria, convergence and accommodation anomalies). Excessive bias or inflexibility of response to cues may result in clinically significant problems. Consideration of bias toward blur or disparity as drives for accommodation and vergence is more useful than AC/A and convergence accommodation to convergence (CA/C) ratios alone. These ratios cannot by themselves explain clinical pictures.

### Why AC/A and CA/C Ratios are Insufficient

AC/A ratios (convergence change driven by a change in blur) are commonly used to explain many characteristics of strabismus, but clinicians may be unaware of significant limitations of clinically measured ratios. For example , 3.0 D of blur is often assumed to drive 3.0 D of accommodation , and so the change in angle per diopter is calculated by dividing by 3—a stimulus (clinical) AC/A ratio. In Figure 1 the upper line (in meter angles (MA) and diopters) represents this “perfect” response (on the y-axis) to the blur produced by near fixation or minus lenses (x-axis). MAs are the vergence equivalent of diopters of accommodation. 1 MA is required to fix at 1 meter and 3 MA are required at 33 cm. They are a useful theoretical measure because they are independent of interpupillary distance (IPD). An infant with an IPD of 45 mm needs 4.5^Δ^ at 1m, whereas a large adult with a 70 mm IPD will converge 7^Δ^ but both will converge 1 MA to fix bifoveally. They are also a useful because MA can be plotted on the same scale as diopters. 3 D of accommodation accompanies 3 MA of convergence, leading to response gains of 1.0 and an AC/A ratio of 1 MA to 1 D (6^Δ^ to 1 D in an adult with an IPD of 60 mm).

**Fig 1:**
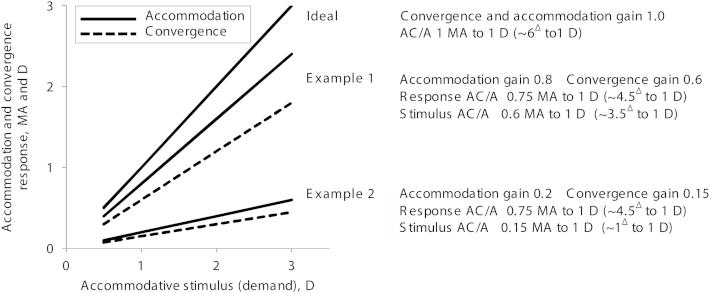
Accommodation and convergence responses to a blur-cue-only target at 2 m, 1 m, 50 cm, and 33 cm, or using −0.5 D, −1.0 D, −2.0 D, and −3.0 D lenses ( 0.5, 1, 2, 3 MA and D demand). Upper (ideal) lines = responses to target demand if the response perfectly matched the stimulus. Lower pairs of responses show two hypothetical participants (examples 1 and 2). Response AC/A ratios are identical, but responses to blur very different. Stimulus AC/A are ratios very different from response ratios, and different from each other. *D*, diopters; *MA*, meter angles.

However, perfect accommodation rarely happens and underaccommodation to blur is common. Figure 1 shows two hypothetical cases, reflecting the range of actual responses we see experimentally. Example 1 accommodates well to an accommodative stimulus and converges slightly less. Example 2 (still typical of many normal people) has lower accommodation and vergence responses to the same blur but still accommodates more than converges. The *stimulus* AC/A ratio (convergence change in relation to the blur stimulus *given*; using 3.0 D as the divisor) will be higher in example 1 because they converge more. The *response* ratios (convergence change in relation to accommodation *response* and the measure that most vision scientists consider the “true” ratio) may be very different. In example 1 the divisor is 2.4 D, but in example 2 it is only 0.6 D; therefore, both stimulus ratios are lower than the response ratios, but *much* lower for example 2. Response ratios rarely correlate with stimulus ratios.^11^ Response ratios tell us the true convergence/ accommodation relationship, but not necessarily how either relate to the stimulus. In both examples, each has an identical “normal” response AC/A ratio of 0.75 MA to 1 D (approximately 4.5^Δ^ to 1 D in an adult), despite very different responses to the change in blur. Example 1 responds well to blur; example 2 does not. Both stimulus and response ratios are missing detail about how we use blur as a cue.

The rarely considered CA/C ratio describes accommodation associated with convergence driven by disparity (rather than vergence to blur above) and is similarly problematical.

The two ratios are inversely,^12^ or reciprocally, related.^1^, ^6^, ^13^, ^14^, ^15^ High AC/A accompanies low CA/C; if blur drives a large amount of convergence, then disparity drives less accommodation. In our laboratory, higher CA/C than AC/A ratios are typical.

In this study we predicted how different biases toward blur and disparity, in combination with anatomical factors, such as position of rest and refractive error, would result in specific diagnoses and treatment responses. We queried our database to determine whether specific diagnoses fitted the model predictions.

## Subjects and Methods

Our dataset was collected from a wide range of normal controls and patients in the course of other published and unpublished studies. Ethics approval was obtained from University of Reading and Berkshire NHS Ethics committees. All subjects provided written informed consent. The clinical details of the different study participants are provided elsewhere.^5^, ^16^, ^17^, ^18^, ^19^, ^20^, ^21^ Subjects fulfilled accepted diagnostic criteria of normality, heterophoria, or intermittent heterotropia, for example, as set out by Ansons and Davis.^22^ All were healthy, 4-42 years of age, with normal binocular vision and stereopsis of at least 120 arcsec at at least one fixation distance; none were amblyopic. We selected the cases by clinical diagnosis, and all cases tested in the laboratory within any diagnosis were included in the analysis; that is, none were rejected because they did not fit our model.

Laboratory data were collected using a remote haploscopic photorefractor to present images moving in depth between distances of 0.33 m to 2 m, described in detail elsewhere^5^ and in e-Supplement 1 (available at jaapos.org). Simultaneous, objective, vergence position and accommodation were calculated from eye position and refraction data collected by a PlusoptiX S04 PowerRefII photorefractor (Plusoptix GmbH, Nuremberg). We calculated convergence angle in MAs and accommodation in D at each fixation distance after correcting for IPD, angle lambda (representing the offset of the corneal reflection from the pupil center, equivalent to angle kappa), and spectacle magnification. We then calculated response gain in relation to demand across the different distances. Different target manipulations allowed us to assess responses to blur, disparity, and proximity separately as the target moved in space. By allowing binocular viewing or by remotely occluding one eye we could present or eliminate disparity. By presenting a detailed clown cartoon or a Gabor patch target we could present or minimize blur cues,^12^, ^23^, ^24^, ^25^and proximal (size/motion/looming) cues could be retained if the same size picture was visible during motion or could be minimized by obscuring target motion and scaling the target for target distance. By varying these conditions, all combinations of the three cues could be tested.

Our discussion concentrates on the three targets most pertinent to our argument: (1) a naturalistic all-cue (*bdp*; blur [*b*], disparity [*d*], and proximal [*p*]) target (detailed clown, viewed binocularly and unscaled for distance); a blur-only (*b*) target (detailed clown, viewed monocularly, scaled-for-distance); disparity only (*d*) target (binocular Gabor patch, also scaled-for-distance).

We calculated response AC/A ratios using the gains in the *b* condition (convergence gain/accommodation gain) and response CA/C ratios using accommodation gain/convergence gain in the *d* condition.

As we argue that the ratios are insufficient, we also needed a measure of bias toward better responses to disparity or blur, independent of the AC/A and CA/C ratios. We averaged vergence and accommodation gains ([accommodation gain + vergence gain]/2) to the *b* target, and those to the *d* target, then subtracted the *b* target gains from the *d* target gains to form a disparity bias index (DBI). If blur and disparity responses are equal, the DBI is zero; a larger positive number indicates a stronger disparity bias, whereas a smaller or negative number indicates more blur bias.

**Fig 2:**
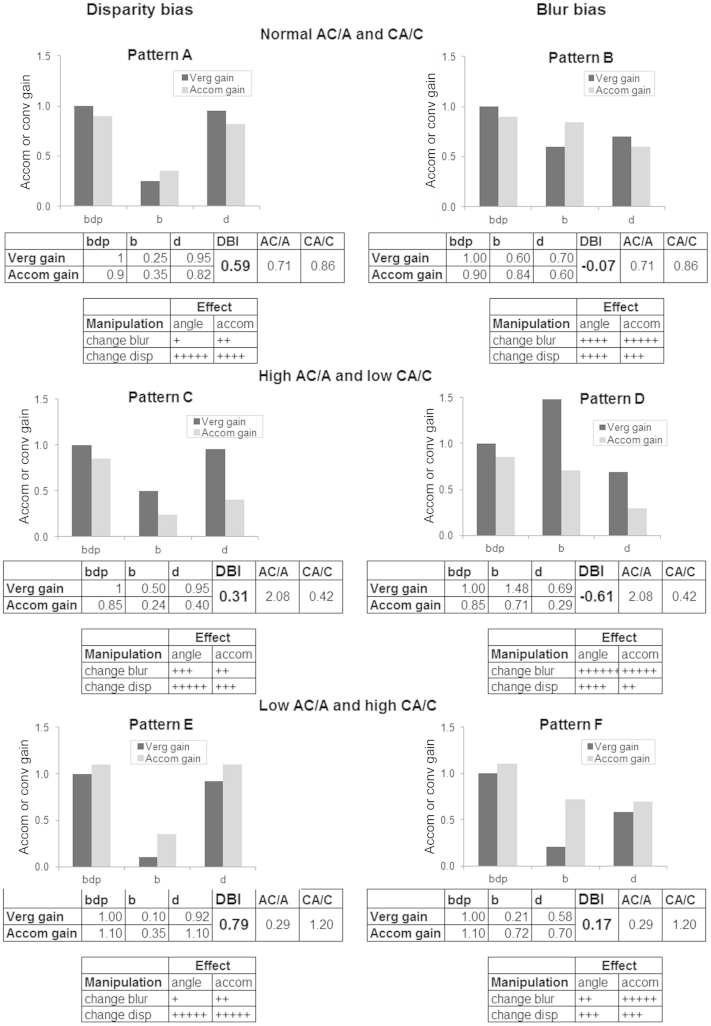
Hypothetical response Patterns A-F. Charts illustrate different response gains to naturalistic (*bdp*), blur-only (*b*) and disparity-only (*d*) cues. Gain of 1.0 = perfect response to cue. Lefthand charts show disparity bias and righthand charts show more blur bias. Below each chart are example values used to calculate AC/A (vergence gain/accommodation gain in the *b* condition), CA/C (accommodation gain / vergence gain in the *d* condition) ratios and Disparity Bias Index (DBI). The tables in each pattern illustrate how treatment to manipulate blur or disparity cues changes the angle of deviation or the accommodation differently. High AC/A and low CA/C ratio response pattern shows vergence responses always exceeding accommodation responses and low AC/A and high CA/C ratio response pattern shows accommodation responses always exceeding vergence responses.
• High AC/A and low CA/C (Figure 2C,D): vergence response gains always greater than accommodation.
• Low AC/A and high CA/C (Figure 2E,F): accommodation response gains always better than convergence.

We present six hypothetical patterns:

• “Normal/classical” AC/A and CA/C (Figure 2A,B): blur driving more accommodation than vergence and disparity driving more vergence than accommodation.

The AC/A and CA/C ratios are identical in each pair, but with different blur and disparity biases. The upper tables under the pairs of charts show the responses used to calculate AC/A and CA/C ratios as well as the disparity bias index (DBI). The lower tables show how changing blur or disparity has different effects on the angle of deviation and/or the accommodation depending on the pattern. Responses to the naturalistic *(bdp)* cue show that overall responses stay within normal limits, but responses to blur and disparity within this stimulus can be very different. We then present real examples drawn from our laboratory dataset (with 95% confidence intervals and statistical tests quoted where large enough numbers permit meaningful analyses). See e-Supplement 1 for details.

## Results

### Normal/Classical AC/A and CA/C Relationships

Patterns A and B show classical normal responses in the naturalistic (*bdp*) condition, with perfect vergence and slight accommodation lag for near (vergence gain, 1.00; accommodation gain, 0.9). Both have identical and normal response AC/A and CA/C ratios, but pattern A's normal responses are stronger to disparity than blur, whereas pattern B's responses to blur and disparity are more equal.

### Pattern A

**Fig 3:**
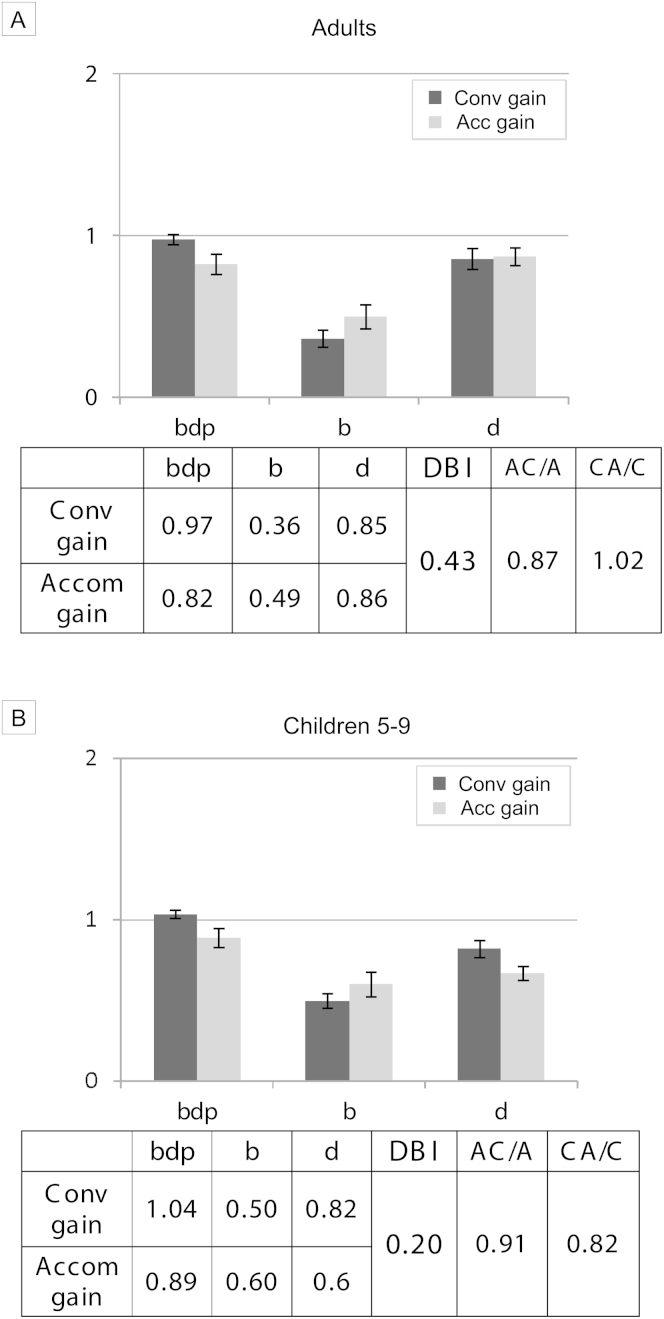
Laboratory data to illustrate normal pattern A. A, Normal adults (n = 56). B, Children 5-9 years of age (n = 24). Both groups show stronger responses to disparity cues similar to Figure 2A. Error bars = 95% CI.

Most normal people are disparity-biased, with more bias in adults (adult (n = 56) DBI 0.43 vs child (n = 24) DBI 0.20, t (78) = 3.52; *P* = 0.001). See Figure 3. Response AC/A ratios are within normal ranges^26^ (approximately 5^Δ^ to 1 D in both groups). The weaker response to blur explains why spectacles rarely change heterophoria much in nonstrabismic individuals. Disparity is the main drive, so changing blur makes little difference if disparity remains available. However, researchers who use naive participants (as opposed to “visual experts,” such as vision scientists and optometry students), consistently find that disruption of binocularity causes a significant drop in accommodation as well as major inaccuracies of convergence that occur in strabismus.^27^, ^28^ Similar findings have been reported in primates.^29^, ^30^

### Pattern B

**Fig 4:**
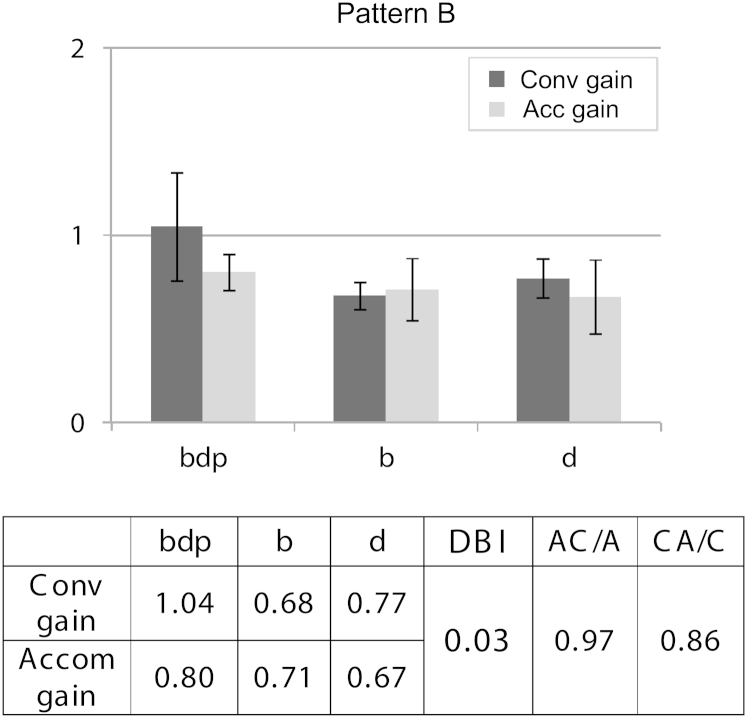
Laboratory data to illustrate pattern B (n = 3). Fully accommodative esotropia with similar near/distance angle of deviation (<8^Δ^ difference with spectacles). Similar responses to those in Figure 3B, with less strong disparity bias. Error bars = 95% CI.

As additional lenses or change in refractive error rarely change angles of deviation in nonstrabismic children and adults, our hypothesis was that disparity bias is normal, whereas accommodative strabismus, where lenses change angles of deviation, would show blur-biased pattern B. As predicted, classic accommodative esotropias (similar near/distance angles; n = 3) followed pattern B (Figure 4), with a low DBI of 0.03, below the lower 95% confidence interval of normal children. Ten further children had higher AC/A accommodative esotropia (near esodeviation more than 10^Δ^ greater than distance while still retaining control with spectacles). These 10 fell between this group and Pattern D below, with a DBI of 0.01 and AC/A ratio of 1.7 MA to 1 D. The vergence *and* accommodation of pattern A are more disrupted by occlusion or suppression disrupting disparity cues but less disrupted by lenses or developing refractive error changing blur. Pattern B is more affected by blur change but less disrupted by disparity change. Because the AC/A and CA/C ratios are normal, blur change drives more accommodation than convergence and disparity change drives more convergence than accommodation.

### Nonclassical Relationships

There are also disparity-biased vs blur-biased alternatives possible for the high AC/A and low CA/C (convergence exceeds accommodation) and the low AC/A and high CA/C (accommodation exceeds convergence) scenarios, each pair with similar ratios (patterns C and D).

### Pattern C

**Fig 5:**
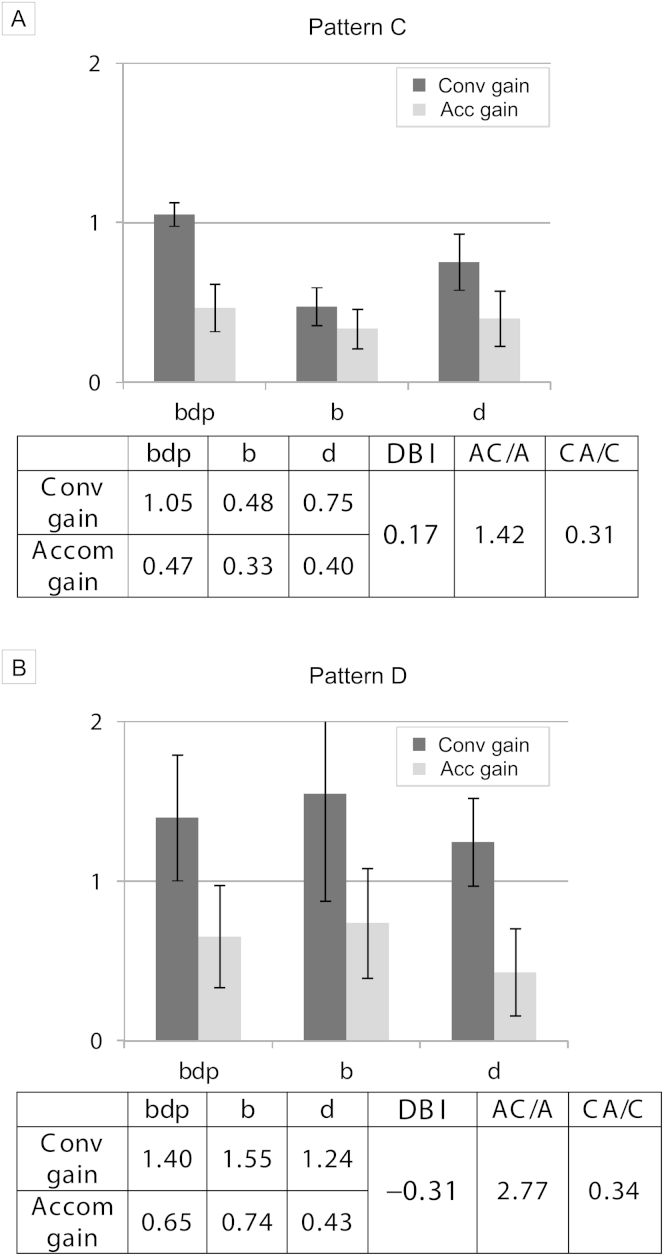
Laboratory data illustrating patterns C (n = 22) and D (n = 8). A, Pattern C: early presbyopia, under 45 years of age. No overconvergence despite high AC/A ratio (approximately 8^Δ^ to 1 D). B, Pattern D: high AC/A ratio convergence excess. This is the only group with naturalistic *(bdp)* vergence gain >1.0, reflecting their overconvergence for near. Note change of y-axis scale and poor accommodation gain in the *bdp* condition. Error bars = 95% CI.

Disparity-biased pattern C shows a higher DBI. Despite the high AC/A ratio, blur stimuli should not lead to overconvergence because blur is a weak cue; underaccommodation occurs, but overconvergence does not. The vergence response to disparity (low CA/C ratio) might not drive sufficient accommodation in the case of orthophoria where there is normal convergence demand, but in exophoria, the additional convergence required to overcome the deviation would not result in overaccommodation. The weak accommodation of early presbyopia (Figure 5A) by necessity means more convergence associated with each unit of accommodation, but overconvergence on attempted near fixation does not occur because disparity, not blur, drives responses. Many well-controlled basic exodeviations, controlling with good accommodation, also fall into this category.

### Pattern D

In blur-biased pattern D the high AC/A ratio causes excessive convergence in response to blur; individuals with this pattern are at risk of convergence excess accommodative esotropia. As predicted, high AC/A ratio convergence excess esotropias (n = 8) fit pattern D, with a negative DBI of −0.31, significantly lower than normal children (t[30] = 2.86; *P* = 0.008). See Figure 5B. These children respond to blur more than normal and so overconverge. Their weaker response to disparity might also explain their original decompensation. Some naturalistic *bdp* responses in the figure show hypoaccommodation, suggesting that some try to control their deviation, but at the expense of clear near vision.

### Low AC/A and High CA/C ratios

Patterns E and F in Figure 2 show that these low AC/A and high CA/C individuals accommodate with relatively little convergence, but when they converge they overaccommodate (also reflected in the slight accommodation lead in the *bdp* condition).

### Pattern E

**Fig 6:**
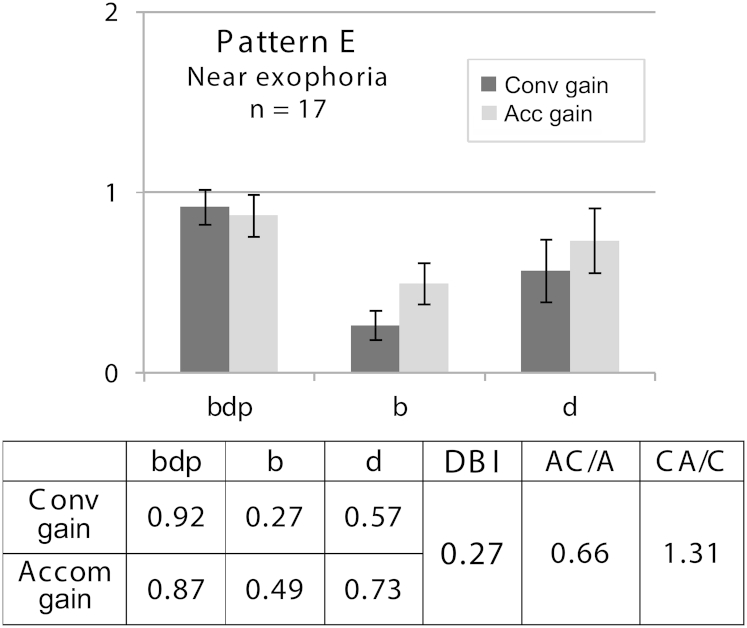
Real laboratory data to illustrate pattern E: adult near exophoria. Error bars = 95% CI.

Disparity-driven pattern E subjects do not accommodate well to increasing blur targets, and do even less convergence, leading to near exophoria. Poor blur response might be found in straight-eyed hyperopic ametropic amblyopia. Reducing disparity cues by dissociation, prisms or surgery, however, significantly reduces accommodation, while the need to control a large exodeviation, for example, might result in *over*accommodation.^9^ Our near exophoric subjects were in a subclinical, asymptomatic group of young adults with small deviations (but with >6^Δ^ larger near angle) and were in fact recruited as normal controls (Figure 6). They had significantly lower AC/A ratios (t [39] = 2.27; *P* = 0.028) than normal and accommodated more than the normal adults while responding to the *d* target (2.98 D vs 2.45 D (t [71] = 3.34); *P* = 0.001). We predict clearer differences in clinical populations.

### Pattern F

Pattern F responds to blur. Although the high CA/C ratio means that accommodation exceeds convergence, the weakness of disparity-driven responses does not result in over-accommodation. We predict that hyperopic children who manage to accommodate over their refractive error without becoming strabismic would fit this group. Our dataset did not include any individuals in pattern F. Our patient participants were drawn from a hospital population, referred for poor visual acuity, so this “silent” group of hyperopic children with good vision is difficult to detect because they pass screening. We predict that subjects with the combination of convergence insufficiency but good accommodation will fit into this group.

## Discussion

We readily accept other theoretical models based on complex feedback loops, engineering-type modeling, AC/A and CA/C cross-links, and slow tonic versus rapid step adaptive mechanisms,^3^, ^4^, ^14^, ^31^, ^32^, ^33^, ^34^ but these models are mainly concerned with subtleties and control of normal behavior. We suggest that consideration of how people use visual cues provides a useful conceptual model for clinicians, who deal with more severe problems outside the envelope of normality. Different blur and disparity biases characterize clinical patterns despite identical AC/A and CA/C ratios.

We suggest clinicians should first consider which aspect of the visual stimulus is mainly used to compute target depth. Is the subject a “disparity person” or a “blur person”? Next, determine whether accommodation and convergence are strongly linked in the individual, assessing changes induced by lenses, prisms, or dissociation, whether the individual accommodates more than converges, or vice versa. AC/A and CA/C ratios help, but are only informative if an individual's blur or disparity bias is also known. How much, or whether, “ratios” matter depends on “style,” basic angle, and refraction. We can then understand etiology and clinical characteristics and predict response to treatment.

Although there are many individual differences in cue use within asymptomatic normal individuals, inflexibility and excessive bias appear to characterize clinical groups and predict treatment responses. Time or orthoptic exercises may alter biases, for example, teaching a blur-biased accommodative esotropic patient to pay closer attention to single vision while accepting some blur.

Disparity people, the majority, maintain binocularity by motor fusion and are less concerned with the effect on accommodation. Lenses change angles little, but they may dissociate more on occlusion. If, as is usual, most of their accommodation is also driven by disparity, then dissociation, prisms or surgery will not only change the angle, but also influence accommodation.

If most people are disparity-driven, why do we not see more accommodative problems? Imprecise accommodation may be normal. Binocular accommodation is better than monocular, monocular accommodation is rarely required, and blur is often tolerated. Our large dataset suggests that hypoaccommodation is common. We found underaccommodation of >1.0 D at 33 cm in 22% of normal adults, 12% of normal children, 22% of hyperopic subjects with glasses, and 100% of hyperopic subjects without glasses.^17^ It is much more common in naive populations than in experienced participants.^10^ Even “accommodative targets” are rarely threshold, that is, smaller than 20/20, or 0.0 logMAR; expert readers decode text from word shape rather than individual letters, while early readers use large print. Precise accommodation is rarely required. Are disparity people also more tolerant of blur and thus happy without their glasses, with suboptimal prescriptions, or with dirty glasses?

Blur people will be more sensitive to changes in clarity and accommodate accordingly. These seem rare among the general population but do appear in our clinical caseload. Even a normal, but especially a high, amount of vergence associated with the response to blur, risks accommodative esotropia in hyperopia as we know. If less vergence is associated with this accommodation (low AC/A), hyperopes may be able to respond to blur without over-converging—an advantage in uncorrected hyperopia, but a disadvantage for the clinician hoping to change an angle with spectacles. Blur-driven people's sensitivity to disparity may be low, so changing an angle with prisms, dissociation or surgery will have less effect on their accommodation but weaker fusion might mean that binocularity is more easily lost. Are these also the people who notice small changes of refractive prescription, be slow to settle into new glasses and insist on them being clean?

Our examples are only illustrative and not comprehensive, but some more predictions from this model are summarized in Table 1.

**Table 1:** Possible categorization of diagnoses and clinical patterns (not exhaustive and some speculative)

	Disparity bias (higher DBI)	Blur bias (lower DBI)
Normal/classical	• Normal • Basic exophoria with accommodation lead • Uncorrected hyperopic—blurred visual acuity but orthophoria; ametropic amblyopia risk if marked • Uncorrected myopic—mild distance blur symptoms but orthophoria • Accommodative consequences from prisms and surgery • “Accommodative stimuli” produce small changes in angle • Few angle changes on refractive correction	• Hyperopic—fully accommodative esotropia or esophoria • Uncorrected myopic—near exophoria; exophoria reduces when corrected • Accommodative changes to angle • Spectacles, or change in refraction, change angle • Fewer changes to accommodation with dissociation or surgery
High AC/A and low CA/C (vergence exceeds accommodation)	• Presbyopia (normal response to disparity—obligatory under-accommodation) • Accommodation insufficiency in orthophoria • Basic exodeviation—well controlled exophoria with normal accommodation • Hyperopic—accommodation insufficiency even when corrected • Few accommodative consequences from surgery or prisms	• Hyperopic—convergence excess esotropia. Need full correction • Myopic—near exophoria as less accommodation drive for near. Exophoria completely resolves on correction • Strong accommodative change in angle, especially for near
Low AC/A and high CA/C (accommodation exceeds vergence)	• Well controlled near exophoria (increase only on dissociation) with normal accommodation • Accommodation for near affected by change in angle / dissociation / prisms • Basic exodeviation—distance exotropia with large accommodation change on dissociation who risk hypo-accommodative convergence excess post-operative esotropia. (we suggest these have been misdiagnosed as high AC/A)^11^ • Uncorrected hyperopic—nonstrabismic but ametropic amblyopia due to excessive near blur ? accommodation insufficiency even when corrected.	• Uncorrected hyperopic—nonstrabismic with good vision; possible accommodation lead when corrected; prefer undercorrection. • Uncorrected myopic—severe blur symptoms and near exophoria (exophoria remains on correction) • Symptomatic convergence insufficiency (poor binocular control of exodeviation for near) with normal accommodation • Refractive correction makes minimal difference to angle

Further laboratory and clinical research is necessary to test these speculations, but in the meantime it is probably clinically important to test clarity of a near target to threshold, to ask about image clarity at every swap of an occluder during a prism cover test and to measure accommodation objectively.

Remaining symptom free may depend on being able to use both blur *or* disparity to drive responses, and to be able accommodate and converge more independently (“positive and negative relative fusion”) to compensate for “style” biases. Our naturalistic paradigm repeatedly finds that responses are variable, accommodation and vergence do not always co-vary, and “ratios” are rarely fixed or repeatable, so perhaps the two systems are, and need to be, less closely linked than we assume.

## Supplementary Data

e-Supplement 1
