# Supplementary material for: Disparity-driven vs blur-driven models of accommodation and convergence in binocular vision and intermittent strabismus
Source: J AAPOS. 2014 Dec;18(6):576–83. doi: 10.1016/j.jaapos.2014.08.009 (PMC4270963; doi:10.1016/j.jaapos.2014.08.009)
Supplement: e-Supplement 1 [file mmc1.pdf]

e-Supplement 1. Data collection method

Apparatus and Testing

The remote haploscopic videorefractor (RHV) presents targets at five fixation distances while collecting continuous recordings of eye position and accommodative response. The method and calibration procedures have been described in detail previously[1] and validation of the targets further described in the online material associated with another paper.[2] It was originally designed so that we could assess very young infants, but we now have a large dataset of recordings from participants of all ages and a wide range of clinical conditions, all tested under identical conditions.

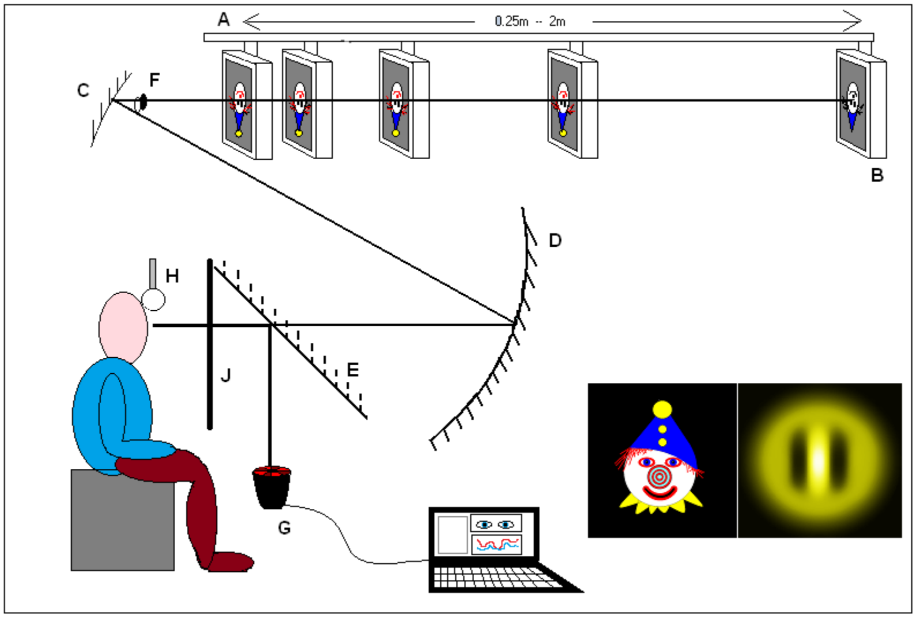

FIG 1. *The remote haploscopic videorefractor. Participants view the target through the upper mirror optical pathway (B,C,D,P) as it moves between 0.25 m and 2.0 m while a PlusoptiX PowerRefII autorefractor measures objective vergence and accommodation via the lower optical pathway (G, E, P). The target appears to be moving directly in front of the participant. A, Motorized beam. B, Video monitor. C & D, Concave mirrors with equal focal distances, creating a virtual image of the participant's eyes at F. E, "Hot" mirror reflects infra-red for*

Copyright 2014 by the American Association for Pediatric Ophthalmology and Strabismus

photorefraction, but allows visible light from target to reach the participant unimpeded; occlusion of half the mirror at F can remotely occlude one eye's view of the target, while still allowing binocular photorefraction. G, Plusoptix PowerRefII photorefractor (infra- red sensor attached to laptop) collects simultaneous refraction and eye position data at 25Hz. H, Headrest. J, Raisable screen can obscure screen during target movement when necessary to minimize looming cues. P, Participant. Two targets – a detailed clown, or blurry Gabor-like target present or minimize blur cues. Both targets can be scaled for target distance if necessary when minimizing looming cues during target movement.

There are two optical pathways designed so that data collection and target presentation can be separated. The eye position and refraction data are collected continuously from each eye at 25Hz via an infra-red "hot" mirror using a PlusoptiXSO4 PowerRefII photorefractor (Plusoptix GmbH,Nurnberg, Germany). The mirror transmits visible light so that the participant has an unimpeded view of the target, but reflects infrared so that the camera sensors can be placed in the same optical plane as the target but without obscuring it. The participants view a monitor screen via two concave mirrors arranged so that the virtual image of the monitor is seen to move backwards and forwards directly in front of the participant (Figure 2). The target image is placed optically at 0.25 m, 0.33 m, 0.5 m, 1 m and 2 m from the participant's eyes, representing response demand of 4, 3, 2, 1 and 0.5 D and meter angles (MA). It moves in a pseudo-random order "run" of 33 cm, 2 m,25 cm,1 m,50 cm, so that a near target is always preceded and followed by a more distant one.

The advantage of using the mirrors for the target presentation pathway is that one eye's view of the target can be occluded remotely by covering half the upper concave mirror in the stimulus pathway. The participants can then only see the target with one eye, but photorefraction of both eyes can still take place via the other optical pathway. Having the occluder remote from the participants' face makes it particularly suitable for use with infants and young children. Typically, approximately one third of older participants in our lab are aware they have been occluded, one third are aware "something has happened to the image" but cannot define what it was, while a further third are completely unaware of the occlusion.

Copyright 2014 by the American Association for Pediatric Ophthalmology and Strabismus

During testing, older infants frequently try to touch the nearest target images, and adults, when asked to try to touch the nearest images at the end of testing, pointed to appropriate point in space, confirming it is a realistic target.

Different targets allow us to manipulate the three main cues to vergence and accommodation. There are eight possible combinations of blur, disparity and proximal/looming cues and typically all eight are presented. Blur cues are available in a detailed cartoon clown target or are minimized by using a Gabor-like image (for validation of these targets see online supplement in Horwood & Riddell 2013).[2] Disparity cues are available when both eyes can see the target or are eliminated by remote occlusion ; proximal looming cues are available when the target can be seen to move and get larger as it approaches or are minimized by putting up a black screen to hide the target as the monitor moves and scaling the target for distance so that it subtends the same visual angle at every distance.

This multiple target paradigm allows us to assess the relative influence of each cue in two ways. Firstly, how much of the response is eliminated by *taking away* the cue, and then how much of the response is driven by that cue in isolation. In adult and older participants the weighting of each cue is broadly similar when manipulated in either way, but in infants and very young children the effect of a cue is more easily seen when it is taken away as single cues drive weak responses.

A typical session in the laboratory involves the participant being tested over 8 runs of the full range of fixation distances. The order of testing was designed so that we could obtain maximum data from infants with limited concentration, where testing might be curtailed at any moment. We have continued to use this testing order so that all the data from our lab is comparable. The first run uses the clown target, binocularly and not scaled so all three cues are presented (*bdp*). The next follows a block of three target runs where one cue is minimized while the others are retained; *bd* – scaled, binocular clown; *bp* – monocular, looming clown; *dp* – binocular, looming Gabor. These three runs are presented in random order. The next block of three runs presents single cues, with the other two minimized ; *b* – monocular, scaled clown ; *d* – binocular, scaled Gabor ; *p*- looming, monocular Gabor, also presented randomly. A final (*o*)target is then presented which minimizes all cues (monocular, scaled, Gabor). This target

Copyright 2014 by the American Association for Pediatric Ophthalmology and Strabismus

enables us to estimate the effect of residual cues we cannot totally eliminate, which seem to be mainly proximal/looming of the dimly visible screen edge. Finally, to assess for fatigue/practice effects the all-cue *bdp* target was presented again. This is followed by a clinical testing session and then the laboratory runs are repeated in a different order with testing blocks.

In terms of this study we wanted to relate our results as closely as possible to the other AC/A and CA/C literature, where experiments are often tightly controlled and extraneous cues excluded, so we have only presented the naturalistic *bdp* target and the *b* and *d* targets. The *b* target allows us to assess the AC/A relationship by measuring how much vergence occurs in relation to accommodation when blur is manipulated. The *d* target allows us to assess the CA/C relationship by measuring how much accommodation occurs in relation to vergence driven by disparity. We have not considered proximal cues here because we and others[1 3] generally find they are very weak in adults with normal binocular vision. We speculate they may be more influential in constant strabismus, as they are in infants[2] and will be reporting these in the future.

We measure diopters of accommodation (D) derived from the refraction calculated by the photorefractor. Vergence is calculated in meter angles (MA). MA are particularly valuable for this type of research as responses can be compared between groups of different ages with widely varying IPD, eg, an infant with an IPD of 45mm will only need 4.5^{Δ} of convergence at 1m, but a large adult with a 70 mm IPD will need 7^{Δ} to 1 D, but both will need 1MA of convergence to be converging appropriately.

Data from the 25 cm (4 D) demand target were discarded due to excessive pupillary constriction preventing collection of many readings, but this target was retained in the testing sequence to maintain the near/distance alternation. We also excluded this target because of the possibility that off-axis differences in peripheral refraction might induce inaccuracy, although at most the vergence angle would only be just over approximately 10°, well within the limits within which peripheral refractive errors are insignificant.[4]

The monitor is moved by a belt powered by a motor outside the apparatus and beyond the farthest target distance at 2.75 m from the participants. While the motor can be heard during target motion, so alerting the participants that movement is occurring, it gives no clues

Copyright 2014 by the American Association for Pediatric Ophthalmology and Strabismus

to the target position or direction of movement. The target screen moves at 0.4 meters per second.

Data Collection and Processing

The tester watched the traces during testing and the target was only moved to the next position in the sequence when traces of both vergence and accommodation could be seen by the tester to have been stable for at least two seconds. Offline, data were converted to vergence (in degrees) and accommodation (in D), and responses were charted against time for the whole run of all five target positions and visually inspected (Figure 5). The macro searched for spikes of data caused by blinks and removed data points immediately before and after them. Representative vignettes of the most stable 25 continuous data points were selected for each target position. Vignettes were only chosen from sections of the data where the response had settled and flattened out for at least 0.5 sec,[5] but before any tonic changes would be expected to have occurred. These responses were averaged, and the accommodative and vergence planes were calculated using a macro developed in our laboratory which uses raw data to correct for individual angle lambda and inter-pupillary distance (IPD), any spectacle maginfication and a systematic error in increasing underestimation of accommodation in comparison to dynamic retinoscopy found during earlier calibration studies (using the formula $1.2385x+0.799$, where x equals the PlusoptiXS04 accommodation measure).[1] We obtained the best estimate of angle lambda when fixing at infinity by plotting the y-intercept of the nasal displacement from the pupil center averaged across both eyes at all four fixation distances in the all cue (*bdp*) condition. True IPD was calculated from the y-intercept of PlusoptixS04 IPD plotted against target distance at all four fixation distances in the *bdp* condition. This was used to calculate responses in MA for each participant. Individual accommodation calibration was not carried out in view of the long testing session for very young infants, but other studies using similar apparatus have shown that group means in infants and adults are similar.[6,7] Interscorer reliability on masked scoring where each scorer was free to choose the vignette was excellent. For both vergence and accommodation, this analysis showed a high agreement: for vergence: $r = 0.99$, mean interscorer difference = 0.037 ± 95% CI 0.37 MA; for accommodation $r = 0.99$, mean interscorer difference 0.0095 ± 95% CI 0.175 D.

Copyright 2014 by the American Association for Pediatric Ophthalmology and Strabismus

Clinical Data

We have collected a large data series of different clinical diagnoses in the laboratory, all tested under identical conditions. Patient participants were recruited from a hospital population and all were wearing up-to-date spectacles, if required, prescribed from cycloplegic refraction. Every participant also received a full orthoptic investigation on their visit to the laboratory, so that we could ensure test instructions and lighting conditions were controlled.

Clinical diagnoses and data are kept on a separate database from the laboratory data (which is pseudonymized and filed by number only). For this study we made prior hypotheses about which diagnosis would fit which of the six scenarios driven by predictions of the model (A-F). We then included for analysis and presentation all cases from our diagnosis database with that clinical diagnosis, that is, we did not select them because they had a particular bias, or exclude them if they did not fit the pattern. As our research focus has been in some specific areas, we have larger numbers in some categories than others, so group sizes are sometimes small and illustrative, rather than definitive and large enough for statistical analysis.

We used orthoptic diagnostic criteria according to Ansons and Davis.[8] Diagnoses were made on the basis of the conditions which determined control of deviation (eg, manifest with/without correction, near /distance control and angle) and supplemented with information from prism cover tests in the case of the exophorias. All had equal and normal visual acuity (0.0 logMAR in each eye), bifoveal binocular fixation at some distance or with refractive correction such as bifocals, with 120" arc stereopsis using the TNO test, and no suppression response on the 4^{Δ} BO prism test. None had had treatment for amblyopia. Apart from the early presbyopes, all had clinically normal accommodation with near points of at least 8 cm. Angles of deviation were measured with prism cover tests.

The early presbyopes were drawn from staff in the University. The near exotropia group were not a clinical sample. They had been found incidentally during recruitment of normal controls and were asymptomatic. They all had near exodeviations of $>6^{\Delta}$ (mean, $8.^{8\Delta}$ 95% CI +/- 1.1^{Δ}) and $<2^{\Delta}$ exophoria in the distance.

References

Copyright 2014 by the American Association for Pediatric Ophthalmology and Strabismus

1. Horwood A, Riddell P. The use of cues to convergence and accommodation in naïve, uninstructed participants. Vision Research. 2008;48(15):1613-1624.
2. Horwood A, Riddell P. Developmental changes in the balance of disparity, blur and looming/proximity cues to drive ocular alignment and focus. Perception. 2013;42:693-715.
3. Wismeijer DA, Erkelens CJ. The effect of changing size on vergence is mediated by changing disparity. J Vis. 2009;9(13):12 11-10.
4. Calver R, Radhakrishnan H, Osuobeni E, O'Leary D. Peripheral refraction for distance and near vision in emmetropes and myopes. Ophthalmic Physiol Opt. Nov 2007;27(6):584-593.
5. Tondel GM, Candy TR. Human infants' accommodation responses to dynamic stimuli. Invest Ophthalmol Vis Sci. Feb 2007;48(2):949-956.
6. Bharadwaj S, Sravani N, Little J-A, et al. Empirical variability in the calibration of slope-based eccentric photorefraction. J Opt Soc Am A. 2013;30(5):923-931.
7. Blade PJ, Candy TR. Validation of the PowerRefractor for measuring human infant refraction. Optom Vis Sci. Jun 2006;83(6):346-353.
8. Ansons A, Davis H. Diagnosis and management of ocular motility disorders. Oxford: Blackwell Science; 2001.

Copyright 2014 by the American Association for Pediatric Ophthalmology and Strabismus
